# Goitre

**Published:** 1890-03-01

**Authors:** 


### GOITRE.

Mr. James Berry recently brought the subject of goitre before the Medical Society of London, illustrated by specimens, photographs, etc. In the earlier stages of the disease there is general hypertrophy of the whole gland, attended with colloid secretions in the thyroid vesicles. Afterwards cysts may form. So-called fibrous goitre is more uncommon than is supposed. Neither is the gland so vascular as has been supposed. Three photographs showed stages of cachexia strumipriva produced by the total extirpation of the gland. In order to avoid this cachexia, Mr. Horsley proposed a monkey's thyroid should be transplanted. Sir J. Fayrer coupled goitre and so-called malarious anæmia. Mr. Berry doubted the efficacy of transplantation, and was unable to recognise the connexion between malarious spleen and goitre, for they often occurred separately. Although the treatment by biniodide of mercury ointment was mentioned during the discussion, Sir Joseph Fayrer did not speak of it with fervour, saying although it gave relief he did not get many cures. As a matter of fact the treatment of goitre is not very encouraging. Ligature of arteries connected with the enlarged gland has not proved successful. The same may be said of injections of various substances into the enlarged tissue, which have been tried and found wanting. And the extirpation of the gland being so frequently followed by cachexia strumipriva as almost to forbid this operation. Goitre, as is well known, is endemic in certain localities, although opinions are divided as to the cause of the malady. In our opinion, unless the goitre causes danger to life by pressure on the trachea or otherwise, it is best to avoid surgical treatment. In the meantime the most successful plan is removal from the endemic locality. This measure, together with iodide of potassium internally and biniodide ointment externally will often cure simple goitre of recent origin. Operative interference is rarely justifiable, as the danger from hæmorrhage, wounds of the trachæa, wounds of large nerve trunks, and entrance of air into the veins are great.

